# MXene Boosted CoNi-ZIF-67 as Highly Efficient Electrocatalysts for Oxygen Evolution

**DOI:** 10.3390/nano9050775

**Published:** 2019-05-20

**Authors:** Yangyang Wen, Zhiting Wei, Chang Ma, Xiaofei Xing, Zhenxing Li, Dan Luo

**Affiliations:** State Key Laboratory of Heavy Oil Processing, College of New Energy and Material, Beijing Key Laboratory of Biogas Upgrading Utilization, China University of Petroleum (Beijing), Beijing 102249, China; WZT1215385749@126.com (Z.W.); machang_cup@126.com (C.M.); xingxiaofei_cup@126.com (X.X.)

**Keywords:** oxygen evolution reaction, metal-organic frameworks, MXene, Ti_3_C_2_T_x_, hybrid

## Abstract

Oxygen evolution reaction (OER) is a pivotal step for many sustainable energy technologies, and exploring inexpensive and highly efficient electrocatalysts is one of the most crucial but challenging issues to overcome the sluggish kinetics and high overpotentials during OER. Among the numerous electrocatalysts, metal-organic frameworks (MOFs) have emerged as promising due to their high specific surface area, tunable porosity, and diversity of metal centers and functional groups. It is believed that combining MOFs with conductive nanostructures could significantly improve their catalytic activities. In this study, an MXene supported CoNi-ZIF-67 hybrid (CoNi-ZIF-67@Ti_3_C_2_T_x_) was synthesized through the in-situ growth of bimetallic CoNi-ZIF-67 rhombic dodecahedrons on the Ti_3_C_2_T_x_ matrix via a coprecipitation reaction. It is revealed that the inclusion of the MXene matrix not only produces smaller CoNi-ZIF-67 particles, but also increases the average oxidation of Co/Ni elements, endowing the CoNi-ZIF-67@Ti_3_C_2_T_x_ as an excellent OER electrocatalyst. The effective synergy of the electrochemically active CoNi-ZIF-67 phase and highly conductive MXene support prompts the hybrid to process a superior OER catalytic activity with a low onset potential (275 mV vs. a reversible hydrogen electrode, RHE) and Tafel slope (65.1 mV∙dec^−1^), much better than the IrO_2_ catalysts and the pure CoNi-ZIF-67. This work may pave a new way for developing efficient non-precious metal catalyst materials.

## 1. Introduction

With the rapid combustion of fossil fuels and the ever-growing concerns relating to the environmental crisis, developing sustainable energy technologies (such as metal-air batteries and water splitting) has triggered extensive attention [1]. Oxygen evolution reaction (OER) is the key process for these electricity-driven devices, but it has been significantly hindered by its sluggish kinetics and substantial overpotential [2]. Therefore, highly active electrocatalysts are required to increase the reaction rate and to lower the overpotentials in the OER process. To date, the precious metal oxides (e.g., RuO_2_ and IrO_2_) are the best electrocatalysts with a promoted proton-coupled charge transfer process, but their scale-up implementation has been greatly hampered by their high price, scarcity and poor durability [3,4]. Within this context, increasing efforts have been devoted to the exploration of inexpensive, earth-abundant and highly efficient electrocatalysts for OER [5]. Among them, the earth-based transition metal-rich compounds, including transition metal oxides [3], sulfides [6] and phosphides [7], have exhibited great promise as OER electrocatalysts.

Recently, metal organic frameworks (MOFs) consisting of the coordination of organic ligands and metal ions or clusters have received increasing attention for catalysis-related applications [4,8]. MOFs are an important class of porous solids in electrocatalysis in view of their high specific surface area, tunable porosity, and diversity of metal centers and functional groups [9]. However, it is still a challenge to directly utilize MOFs as efficient OER electrocatalysts because of their poor conductivity. One commonly-used strategy is adopting MOFs as precursors to prepare metal-based compounds/porous carbon composites via a high-temperature pyrolysis. However, the active sites and intrinsic structure of MOFs are inevitably sacrificed with the loss of organic ligands during the pyrolysis [10]. Another possible strategy is to combine MOFs with conductive nanostructures, which has demonstrated a significant enhancement in the electrocatalytic properties [11,12].

MXene is a new class of two-dimensional materials, synthesized by selectively etching A layers from its MAX phase [13]. It can be represented using a formula of M_n+1_X_n_T_x_, where M is the early transition metal, X stands for C and/or N elements, and T for the surface terminations (–O, –F or –OH) [14]. MXene has emerged as a promising nanomaterial in various fields, including energy storage [15,16], energy conversion [11,17], water purification [18], electromagnetic interference [19], and so on, owing to its excellent electrical conductivity and surface hydrophilicity. Besides, MXene also demonstrated the possibility of being an excellent support by altering the electrophilicity of active centers in the supported catalysts and thus modifying the catalytic activity of the composites [20].

In this work, a MXene supported CoNi-ZIF-67 hybrid (CoNi-ZIF-67@Ti_3_C_2_T_x_) was synthesized by the in-situ growth of bimetallic CoNi-ZIF-67 rhombic dodecahedrons on the Ti_3_C_2_T_x_ matrix via a coprecipitation reaction. The effective synergy of the CoNi-ZIF-67 and MXene phases endows the hybrid with a remarkable electrocatalytic activity for OER, with a low onset potential (275 mV vs. a reversible hydrogen electrode, RHE) and Tafel slope (65.1 mV∙dec^−1^).

## 2. Materials and Methods

### 2.1. Preparation of Ti_3_C_2_T_x_ MXene

Ti_3_AlC_2_ powder was first prepared via the HF-etching method [21]. Briefly, 1 g of Ti_3_AlC_2_ powder was blended with 40 mL of 40 wt.% HF solution under continuous stirring at 45 °C for 24 h. The resulting suspension was separated by centrifugation, washed several times with distilled water, and freeze-dried, obtaining the accordion-like Ti_3_C_2_T_x_ MXene.

### 2.2. Preparation of CoNi-ZIF-67@Ti_3_C_2_T_x_ and Pure CoNi-ZIF-67

Typically, 300 mg Ti_3_C_2_T_x_, 0.9 mmol Co(NO_3_)_2_·6H_2_O and 0.1 mmol Ni(NO_3_)_2_∙6H_2_O were dispersed in 8 mL methanol under sonication for 1 h. Then, 8 mmol of 2-methylimidazole was dissolved in another 8 mL methanol under stirring for 30 min. The two above solutions were mixed together, followed by adding 2 mg of hexadecyl trimethyl ammonium bromide (CTAB) and continuously stirring for 8 h at room temperature. The final precipitates were collected by centrifugation, washed with methanol and water several times, and dried at 60 °C under vacuum for 12 h. As a control, pure CoNi-ZIF-67 was prepared via the same procedure but without adding the Ti_3_C_2_T_x_.

### 2.3. Materials Characterizations

The morphology and structure of the as-prepared catalysts were characterized by scanning electron microscopy (SEM, Hitachi SU8010, Tokyo, Japan), transmission electron microscopy (TEM, JEM 2100 LaB6, Tokyo, Japan), powder X-ray diffractometer analysis (XRD, Bruker D8 Advance instrument, Karlsruhe, Germany) with a Cu Kα irradiation source at a scanning rate of 1° per min, and X-ray photoelectron spectroscopy (XPS, PHI5000 Versaprobe, Kanagawa, Japan) with an Al Kα X-ray source. The binding energies of the XPS measurements were calibrated to the C 1s peak at 285.0 eV. The specific surface areas and pore size distribution of the catalysts were conducted on the ASAP2460 Surface Area and Porosity Analyzer (Micromeritics, Atlanta, GA, USA). The surface areas (*S*_BET_) were calculated from the N_2_ sorption isotherms via the Brunauer-Emmett-Teller method, and the pore size distributions were calculated from the N_2_ isotherms using the non-local density functional theory (NLDFT) method.

### 2.4. Electrode Preparation and Electrochemical Measurements

All electrocatalytic performances were evaluated on a CHI 760E electrochemical workstation (Chenhua Instrument, Shanghai, China) with a standard three-electrode system in 0.1 M KOH aqueous solution at room temperature. A glassy carbon electrode (GCE, 5 mm in diameter) coated with the as-prepared catalysts was employed as the working electrode, a Hg/HgO electrode as the reference electrode and a graphite rod as the counter electrode. Before the test, the catalyst ink was prepared by dispersing 10 mg of catalyst powder in a mixture of 40 μL 5 wt.% Nafion solution (Sigma-Aldrich, Shanghai, China), 750 μL water and 250 μL ethanol. After ultrasonication for 30 min, 10 μL of the catalyst ink was pipetted onto the freshly-polished GCE with a catalyst mass loading of ~0.5 mg_cat_∙cm^−2^. All the potentials were calibrated to a reversible hydrogen electrode (RHE) according to the equation, *E*(RHE) = *E*(Hg/HgO) + 0.059pH + 0.098. Before the electrochemical measurement, the electrolyte was bubbled with an O_2_ flow for 30 min, and a gas flow was maintained over the electrolyte during the measurement to ensure the O_2_ saturation. The polarization curves were tested using the linear sweep voltammetry (LSV) at a scan rate of 50 mV∙s^−1^. The double-layer capacitance (*C*_dl_) was calculated from the cyclic voltammetry (CV) curves in a small potential range of 1.023–1.073 V vs. RHE without the occurrence of an apparent faradic process. The plots of the current density difference [Δ*J* = (*J*a − *J*c)], at 1.048 V vs. RHE against the scan rates of 10–60 mV∙s^−1^, were linearly fitted, and the slope is the *C*_dl_ of the catalysts. Electrochemical impedance spectroscopy (EIS) was carried out at 1.46 V vs. RHE in a frequency range of 0.1–10^5^ Hz. For the stability test, the catalysts were performed at 1.46 V vs. RHE over a 20,000 s continuous time. In comparison, the commercial IrO_2_ catalyst purchased from Sigma-Aldrich with the same catalyst mass loading was tested under the same conditions.

## 3. Results

**Figure 1:**
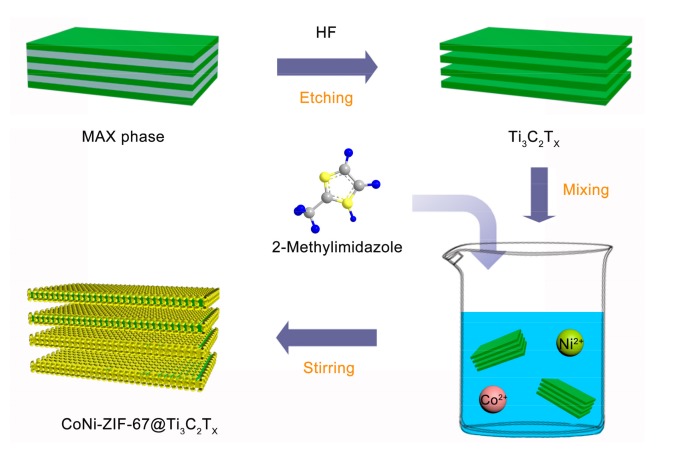
Schematic illustration of the preparation of CoNi-ZIF-67@Ti_3_C_2_T_x_.

The preparation procedure of CoNi-ZIF-67@Ti_3_C_2_T_x_ is illustrated in Figure 1. In brief, the accordion-like Ti_3_C_2_T_x_ was first prepared by the selective etching of Al layers from the Ti_3_AlC_2_ MAX phase using HF. Then, Co^2+^ and Ni^2+^ ions with 2-methylimidazole in methanol were added. The bimetallic CoNi-ZIF-67 could grow in-situ on the Ti_3_C_2_T_x_ via a coprecipitation reaction. Considering the negatively charged Ti_3_C_2_T_x_ surface due to the presence of numerous surface termination groups (e.g., –O, –OH, and –F) introduced during the etching process, Co^2+^ and Ni^2+^ ions could be easily adsorbed on these termination group sites by electrostatic interaction, and could in-situ synthesize CoNi-ZIF-67 rhombic dodecahedrons on the surface and between the interlayers of Ti_3_C_2_T_x_.

**Figure 2:**
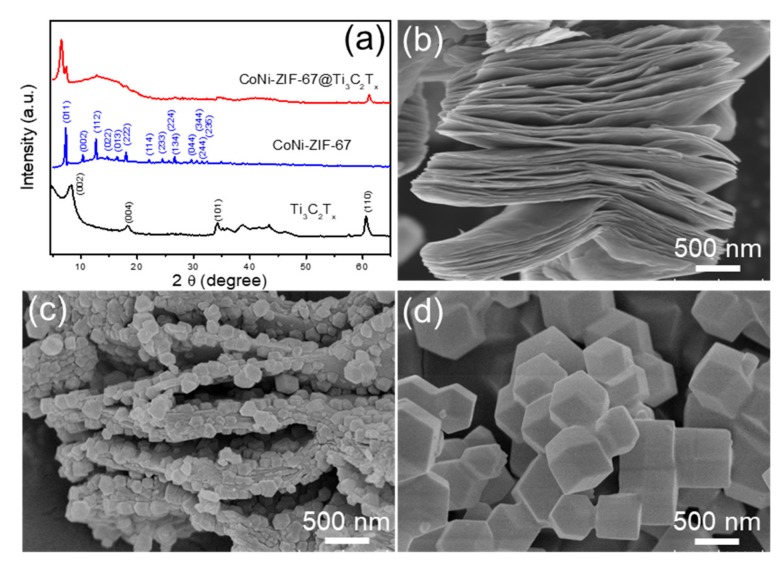
(**a**) X-ray diffraction (XRD) patterns of Ti_3_C_2_T_x_, CoNi-ZIF-67@Ti_3_C_2_T_x_, and pure CoNi-ZIF-67. Scanning electron microscopy (SEM) images of (**b**) Ti_3_C_2_T_x_; (**c**) CoNi-ZIF-67@Ti_3_C_2_T_x_, and (**d**) pure CoNi-ZIF-67.

The XRD analysis was investigated for the structural characterization of the pristine Ti_3_C_2_T_x_, CoNi-ZIF-67@Ti_3_C_2_T_x_, and pure CoNi-ZIF-67. As shown in Figure 2a (enlarged image in Figure S1), the XRD pattern of the pristine Ti_3_C_2_T_x_ represents the characteristic strong peak of the (002) plane at 8.2°, and the weak peaks of the (004), (101) and (110) planes according to the JCPDS card no. 52-0875, confirming the successful preparation of the Ti_3_C_2_T_x_ phases. The pure CoNi-ZIF-67 shows the typical sharp peaks, consistent with the reported literatures [22]. The XRD pattern of the CoNi-ZIF-67@Ti_3_C_2_T_x_ hybrid displays a superimposition of the two phases, featured with four obvious peaks at 6.6° of the (002) plane and 61.2° of the (110) plane for Ti_3_C_2_T_x_, and 7.4° of the (011) plane and 12.8° of the (112) plane for CoNi-ZIF-67, revealing the effective combination of the CoNi-ZIF-67 and Ti_3_C_2_T_x_ phases. It notes that an apparent shift of the (002) plane to a lower angle was detected in the CoNi-ZIF-67@Ti_3_C_2_T_x_, compared with the pristine Ti_3_C_2_T_x_ phase. This left-shift suggests a c-lattice parameter change from 2.16 nm in Ti_3_C_2_T_x_ to 2.68 nm in the hybrid, disclosing the intercalation of the Ti_3_C_2_T_x_ layers due to the inclusion of the CoNi-ZIF-67 particles.

The morphology of the as-prepared catalysts was characterized by SEM and TEM, revealing the hybrid structure of CoNi-ZIF-67@Ti_3_C_2_T_x_. Figure 2b shows the SEM image of the pristine Ti_3_C_2_T_x,_ showing the typical accordion-like structure. The CoNi-ZIF-67@Ti_3_C_2_T_x_ hybrid remains a multilayered structure as the pristine Ti_3_C_2_T_x_ but attached with numerous small particles with a size of 100–200 nm between the interlayers of Ti_3_C_2_T_x_ (Figure 2c). During the HF etching process, Al layers were removed from the MAX phase, and Ti atoms were bonded with the surface functional groups (–O, –OH, or –F), conferring the Ti_3_C_2_T_x_ with negatively charged surfaces [23], which would facilitate the absorption of positively charged Co^2+^ and/or Ni^2+^ ions and subsequently coordinate with 2-methylimidazole molecules for an in-situ synthesis of the CoNi-ZIF-67 particles [11]. As a result, a significant intercalation of the Ti_3_C_2_T_x_ layers is observed in Figure 2c due to the inclusion of the CoNi-ZIF-67 particles. Figure 2d presents the SEM image of the pure CoNi-ZIF-67, showing the typical rhombic dodecahedral structure of CoNi-ZIF-67 and a uniform particle size of 400–600 nm. Particularly, the CoNi-ZIF-67 particles grown on the Ti_3_C_2_T_x_ surface are much smaller than the pure CoNi-ZIF-67 particles (Figure 2c,d). It is speculated that the Ti_3_C_2_T_x_ matrix reduced the aggregation of CoNi-ZIF-67 and deterred the particle growth of CoNi-ZIF-67. In the preparation process, CTAB was added as a sealing agent to aid the formation of smaller and uniform CoNi-ZIF-67 particles. Figure S2 shows the SEM images of two CoNi-ZIF-67 catalysts with CTAB and without CTAB. It can be seen that the CoNi-ZIF-67 particles using CTAB are in the range of 400–600 nm (Figure S2a,b), while the CoNi-ZIF-67 without CTAB exhibits a relatively wider particle size range, from 300 nm to 1 μm (Figure S2c,d). Besides, it is revealed that the CTAB could also facilitate the intercalation of the CoNi-ZIF-67 particles into the interlayers of Ti_3_C_2_T_x_ (Figure S3).

**Figure 3:**
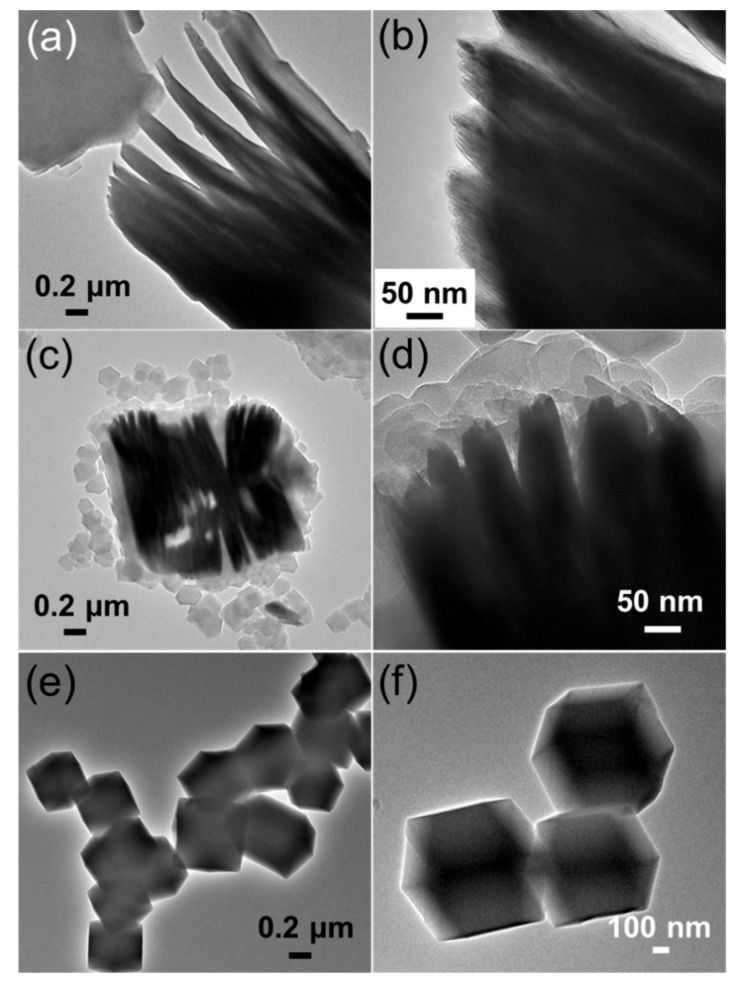
Transmission electron microscopy (TEM) images of (**a**,**b**) Ti_3_C_2_T_x_, (**c**,**d**) CoNi-ZIF-67@Ti_3_C_2_T_x_, and (**e**,**f**) pure CoNi-ZIF-67 at different magnifications.

The TEM images in Figure 3 confirm the hybrid structure of CoNi-ZIF-67@Ti_3_C_2_T_x_ and the rhombic dodecahedral structure of pure CoNi-ZIF-67. Compared with the accordion-like structure of Ti_3_C_2_T_x_, the composite maintained the multilayered structure but was firmly attached with numerous particles on the surface and between the interlayers of Ti_3_C_2_T_x_. It should be noted that the pristine MXene shows obvious lattice fringes for the layers (Figure 3b), while the lattice fringes were not observed in the CoNi-ZIF-67@Ti_3_C_2_T_x_ (Figure 3d). We speculate that the MXene is coated with a thick layer of CoNi-ZIF-67 in the hybrid, and that it is therefore not as easy to observe the lattice fringes in the hybrid as in the pristine MXene. In addition, the high-angle annular dark-field scanning transmission electron microscopy (HAADF-STEM) and EDX elemental mapping images of the CoNi-ZIF-67@Ti_3_C_2_T_x_ hybrid demonstrate the distribution of C, Co and Ni elements on the surface of MXene (Figure S4).

The porosity of the as-prepared catalysts was measured by nitrogen adsorption isotherms, as shown in Figure S5a. The specific surface areas (*S*_BET_) for Ti_3_C_2_T_x_, CoNi-ZIF-67@Ti_3_C_2_T_x_, and pure CoNi-ZIF-67 were 14.1, 202.9, and 1135.8 m^2^∙g^−1^, respectively. The pure CoNi-ZIF-67 exhibits a dominant pore size of 1.08 and 1.3 nm, while the CoNi-ZIF-67@Ti_3_C_2_T_x_ provides a larger dominant pore size of 1.74 nm (Figure S5b–d).

**Figure 4:**
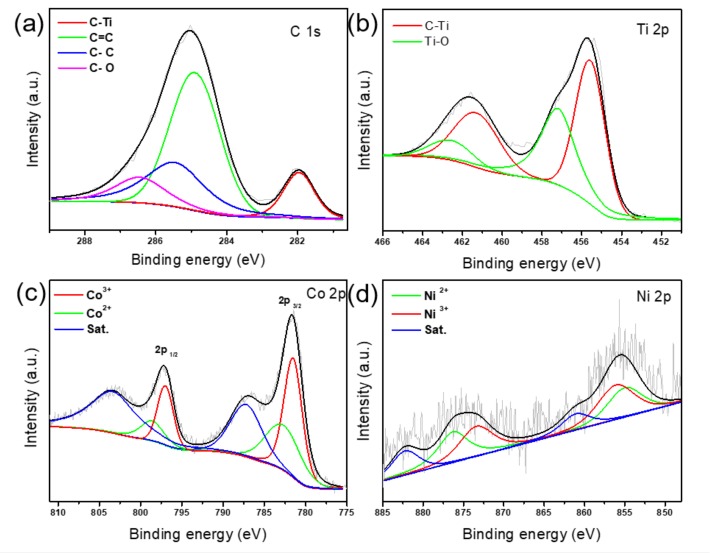
High resolution X-ray photoelectron spectroscopy (XPS) spectrum of (**a**) C 1s; (**b**) Ti 2p; (**c**) Co 2p and (**d**) Ni 2p for CoNi-ZIF-67@Ti_3_C_2_T_x_.

The XPS analysis further confirms the co-existence of C, Ti, Co and Ni elements in the CoNi-ZIF-67@Ti_3_C_2_T_x_, with the elemental contents of 50.4, 11.2, 2.9 and 0.3 at.%, respectively (Table S1). The high resolution C 1s spectrum in Figure 4a can be deconvoluted into four peaks at 282.0, 285.0, 285.5 and 286.5 eV, which are attributed to the C–Ti, C=C, C–C and C–O species [11], respectively. The Ti region shows two pairs of 2p_3/2_/2p_1/2_ doublets for the Ti–C (455.6 eV) and Ti–O (457.3 eV) species [21]. The Co 2p spectrum features three prominent species: Co^2+^ (782.6 eV for 2p_3/2_), Co^3+^ (781.5 eV for 2p_3/2_) and satellite (787.2 eV) [24]. The MXene has an abundant number of surface termination groups (e.g., –O, –OH, and –F), which could adsorb the Co^2+^/Ni^2+^ ions on the MXene surface and may change the Co/Ni oxidation during the pyrolysis process in the inert atmosphere. Consequently, the high-resolution Co/Ni XPS fitting may be helpful for explaining this part. The noise of the Co/Ni region is relatively high due to their low concentrations (Figure 4c,d). Consequently, we fitted the XPS data within the fitting error ($\sum^{}\chi^{2}$) below 2. The Ni 2p region was analyzed into Ni^2+^ (855.0 eV for 2p_3/2_), Ni^3+^ (856.7 eV for 2p_3/2_) and satellite (861.1 eV) [25]. The core level peak analyses for the Co and Ni elements were listed in Tables S2 and S3. Interestingly, the CoNi-ZIF-67@Ti_3_C_2_T_x_ exhibits a relatively higher ratio for the Co^3+^/Co^2+^ species than the pure CoNi-ZIF-67 does, and the same trend can be observed in the Ni elements (Figure S6). We speculate that the introduction of MXene leads to the oxidation of the Co and Ni species in the CoNi-ZIF-67 phases, which may result from the numerous surface terminations on the MXene (–O or –OH), and which thus indicates the interaction between the MXene substrate and the in-situ grown CoNi-ZIF-67 phases.

**Figure 5:**
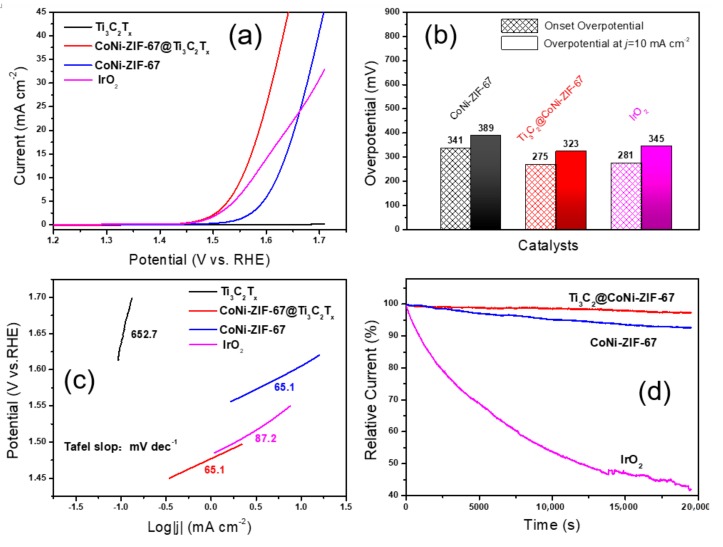
(**a**) Linear sweep voltammetry (LSV) curves of the catalyst Ti_3_C_2_T_x_, CoNi-ZIF-67@Ti_3_C_2_T_x_, pure CoNi-ZIF-67 and IrO_2_ at 50 mV∙s^−1^; (**b**) A comparison of the catalysts in the onset potential and overpotential at a current density of 10 mA∙cm^−2^; (**c**) Tafel plots of the different catalysts; and (**d**) Chronoamperometry curves at 1.46 V vs. RHE over a 20,000 s continuous time.

The electrocatalytic activity of the as-prepared catalysts was first evaluated in a 0.1 M KOH solution in a standard three-electrode cell. Figure 5a presents the iR-corrected linear sweep voltammetry (LSV) curves at a scan rate of 50 mV∙s^−1^. It is apparent that the Ti_3_C_2_T_x_ has no OER activity. Meanwhile, the CoNi-ZIF-67@Ti_3_C_2_T_x_ hybrid shows an enhanced electrocatalytic activity with a much larger current density than the pure CoNi-ZIF-67, which confirms the positive effect of the Ti_3_C_2_T_x_ matrix on enhancing the OER activity. Accordingly, the CoNi-ZIF-67@Ti_3_C_2_T_x_ displays a lower onset overpotential of 275 mV than the pure CoNi-ZIF-67 does (341 mV). The OER activity is also better than that of the as-purchased IrO_2_ catalyst, with an onset potential of 281 mV, which indicates the good electrocatalytic performance of the CoNi-ZIF-67@Ti_3_C_2_T_x_ hybrid. Another critical indicator of the OER activity is the overpotential at a current density of 10 mA∙cm^−2^ (η_j = 10_), which is generally attributed to an approximately 10% efficient solar-to-fuel conversion device [23]. As listed in Figure 5b, the CoNi-ZIF-67@Ti_3_C_2_T_x_ shows the lowest η_j = 10_ value (323 mV), when compared to the CoNi-ZIF-67 (389 mV) and IrO_2_ catalysts (345 mV). It can be seen that the pure CoNi-ZIF-67 exhibited a poor catalytic performance, when compared to the as-purchased IrO_2_ catalyst, which is mainly related to the instinct poor conductivity of the CoNi-ZIF-67, and which thus demonstrates the contribution of the MXene matrix to the good OER activity in the composite. Besides, in view of the XPS analysis (Tables S1–S3), no obvious changes in the Co/Ni atomic ratio, other than an apparent increase in the average oxidation state of both the Co and Ni elements, were detected after introducing the MXene matrix in the CoNi-ZIF-67 phase. We speculated that the enhanced OER activity of CoNi-ZIF-67@Ti_3_C_2_T_x_ may also be related to the altering of the oxidation state of the transmission metal (Co and Ni) active sites [23].

The Tafel slope is a pivotal parameter for providing insightful information on the OER mechanism, particularly for the elucidation of OER kinetics and the rate-determining step [1]. In this regard, the Tafel slopes of the catalysts were plotted in Figure 5c. The value for CoNi-ZIF-67@Ti_3_C_2_T_x_ is 65.1 mV∙dec^−1^, much smaller than that of the as-purchased IrO_2_ catalyst (87.2 mV∙dec^−1^), thus revealing the higher OER rate and favorable kinetics of the CoNi-ZIF-67@Ti_3_C_2_T_x_ hybrid. However, it should be note that, in our work, the Tafel plot of the as-purchased IrO_2_ catalyst is higher than that of the reported nano-sized IrO_2_ catalyst [26], which may be related to the morphology or size of the as-purchased IrO_2_ in the reported works. Additionally, a comparison of the OER performance between the recently reported CoNi-based electrocatalysts with the CoNi-ZIF-67@Ti_3_C_2_T_x_ in this work was listed in Table S4, indicating the excellent electrocatalytic properties of the CoNi-ZIF-67@Ti_3_C_2_T_x_.

Furthermore, the durability of the catalysts was also performed at a constant potential of 1.46 V vs. RHE. As shown in the chronoamperometry curves (Figure 5d), the current of the CoNi-ZIF-67@Ti_3_C_2_T_x_ hybrid remains nearly constant, with up to a 97.3% retention over a continuous time of 20,000 s, which is much more superior than that of the pure CoNi-ZIF-67 (92.6% retention) and that of the IrO_2_ catalyst (only 43.4% remained), demonstrating the excellent stability of the CoNi-ZIF-67@Ti_3_C_2_T_x_.

**Figure 6:**
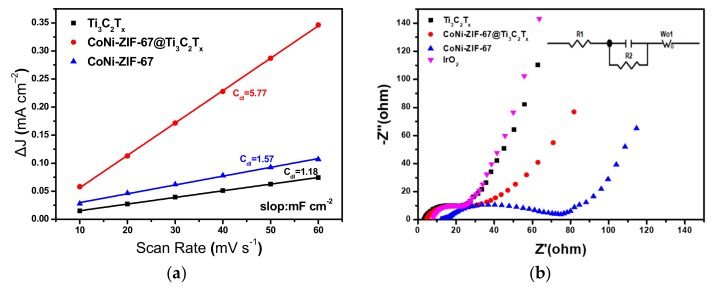
(**a**) Δ*J* = (*J*a−*J*c) plotted scan rates and (**b**) Nyquist plots for different catalysts.

To better understand the catalytic activity of the CoNi-ZIF-67@Ti_3_C_2_T_x_ hybrid, the electrochemically active surface area (ECSA) of the catalysts was investigated. The ECSA is normally positively correlated with the electrochemical double-layer capacitance (*C*_dl_) [27]. Therefore, *C*_dl_ was calculated from the cyclic voltammetry (CV) curves at different scan rates in a narrow potential range of 1.023–1.073 V vs. RHE (Figure S7). As shown in Figure 6a, CoNi-ZIF-67@Ti_3_C_2_T_x_ gives a much higher C_dl_ (5.77 mF∙cm^−2^) than the Ti_3_C_2_T_x_ (1.18 mF∙cm^−2^) and the pure CoNi-ZIF-67 (1.57 mF∙cm^−2^) do, indicating a higher ECSA and more active sites in the hybrid. The higher ECSA is consistent with the smaller particle size of CoNi-ZIF-67 in the hybrid, as shown in Figure 2, which would expose more electrochemical active sites with the electrolyte solution. Additionally, the electrochemical impedance spectroscopy measurement was carried out to analyze the interfacial resistance of the electrocatalysts (Figure 6b). The Nyquist plots of the catalysts were fitted by the RC circuit model, as shown in the inset of Figure 6b, including an internal resistance (R1) and a charge transfer resistance (R2) for the electrochemical reaction [28,29,30,31,32]. The simulated R1 and R2 were shown in Table S5, which reveals that CoNi-ZIF-67@Ti_3_C_2_T_x_ exhibits a smaller R1 and R2 than the pure CoNi-ZIF-67 and IrO_2_, disclosing the optimized charge-transfer capacity of the hybrid during the OER process.

## 4. Conclusions

In summary, a MXene supported CoNi-ZIF-67 hybrid was synthesized via the in-situ growth of CoNi-ZIF-67 rhombic dodecahedrons on the Ti_3_C_2_T_x_ matrix via a coprecipitation reaction. It is shown that the addition of CTAB during the preparation process would aid the formation of smaller and uniform CoNi-ZIF-67 particles, while the CTAB could also facilitate the intercalation of CoNi-ZIF-67 particles into the interlayers of Ti_3_C_2_T_x_, forming a hybrid structure composed of two phases. This CoNi-ZIF-67@Ti_3_C_2_T_x_ hybrid exhibited a superior OER catalytic activity with a low onset potential (275 mV vs. a reversible hydrogen electrode, RHE) and Tafel slope (65.1 mV∙dec^−1^), much better than that of the IrO_2_ catalysts and the pure CoNi-ZIF-67. On the basis of a comprehensive analysis, it is speculated that the good OER activity for the CoNi-ZIF-67@Ti_3_C_2_T_x_ hybrid may be attributed to the following factors: (i) an enhanced conductivity of CoNi-ZIF-67 after the inclusion of the MXene matrix, (ii) a hybrid structure with smaller CoNi-ZIF-67 particles, (iii) an increase in the oxidation state of the Co and Ni elements after the introduction of MXene, and (iv) a high electrochemically active surface area for the hybrid. Therefore, the effective synergy shows the hybrid to be an excellent OER electrocatalyst that may pave a new way for the development of efficient non-precious metal electrocatalysts for OER.

## Supplementary Materials

The following are available online at https://www.mdpi.com/2079-4991/9/5/775/s1, Table S1: Elemental compositions of Catalysts (at.%) determined by XPS, Table S2: Co2p core level peak analyses of catalysts (at.%), Table S3: Ni2p core level peak analyses of catalysts (at.%), Table S4: Comparisons of OER performance between recent reported CoNi-based electrocatalysts with CoNi-ZIF-67@Ti_3_C_2_T_x_, Table S5, The simulated internal resistance (R1) and charge transfer resistance (R2) from the Nyquist plots, Figure S1: Enlarged image of XRD patterns of catalysts, Figure S2: SEM images of pure CoNi-ZIF-67 prepared by the same procedure but with CTAB (a,b) and without CTAB (c,d), Figure S3: SEM image of CoNi-ZIF-67@Ti_3_C_2_T_x_ without using CTAB, Figure S4: HAADF-STEM images and the corresponding elemental maps of C, Ti, Co and Ni in the CoNi-ZIF-67@Ti_3_C_2_T^x^, Figure S5: Nitrogen adsorption isotherms and pore size distribution of catalysts, Figure S6: XPS results of Ti_3_C_2_T_x_ and CoNi-ZIF-67, Figure S7: CV curves in a potential range of 1.023–1.073 V vs. RHE of catalysts.
