# Supplementary material for: MXene Boosted CoNi-ZIF-67 as Highly Efficient Electrocatalysts for Oxygen Evolution
Source: Nanomaterials (Basel). 2019 May 20;9(5):775. doi: 10.3390/nano9050775 (PMC6566882; doi:10.3390/nano9050775)
Supplement: Supplementary file 1 [file nanomaterials-09-00775-s001.pdf]

Electronic Supplementary Information

MXene Boosted CoNi-ZIF-67 as Highly Efficient Electrocatalysts for Oxygen Evolution

Yangyang Wen *, Zhiting Wei, Chang Ma, Xiaofei Xing, Zhenxing Li * and Dan Luo *

State Key Laboratory of Heavy Oil Processing, College of New Energy and Material, Beijing Key Laboratory of Biogas Upgrading Utilization, China University of Petroleum (Beijing), Beijing 102249, China; WZT1215385749@126.com (Z.W.); machang_cup@126.com (C.M.); xingxiaofei_cup@126.com (X.X.)

* Correspondence: wenyangyang@cup.edu.cn (Y.W.); lizx@cup.edu.cn (Z.L.); luodan@iccas.ac.cn (D.L.)

Table S1. Elemental compositions of Catalysts (at.%) determined by XPS.

Catalysts	C	O	N	F	Ti	Co	Ni
$Ti_3C_2T_x$	45.6	17.9	—	18.1	18.4	—	—
CoNi-ZIF-67@$Ti_3C_2T_x$	50.4	13.7	9.0	12.5	11.2	2.9	0.3
CoNi-ZIF-67	69.2	4.8	19.2	—	—	6.1	0.7

Table S2. Co 2p core level peak analyses of catalysts (at.%).

Co2p	Co^{2+} $2p_{1/2}$ (798.1 ± 0.2 eV)	Co^{3+} $2p_{1/2}$ (796.9 ± 0.1 eV)	Satellite (803.2 ± 0.2 eV)	Co^{2+} $2p_{3/2}$ (782.4 ± 0.2 eV)	Co^{3+} $2p_{3/2}$ (781.3 ± 0.2 eV)	Satellite (787.0 ± 0.2 eV)
CoNi-ZIF-67	11.2	7.0	20.3	18.3	14.6	28.6
CoNi-ZIF-67@$Ti_3C_2T_x$	5.5	9.2	26.2	16.0	25.0	18.1

Table S3. Ni 2p core level peak analyses of catalysts (at.%).

Ni2p	Ni^{2+} $2p_{1/2}$ (876.1 ± 0.2 eV)	Ni^{3+} $2p_{1/2}$ (873.3 ± 0.1 eV)	Satellite (882.9 ± 0.4 eV)	Ni^{2+} $2p_{3/2}$ (855.0 ± 0.1 eV)	Ni^{3+} $2p_{3/2}$ (856.7 ± 0.1 eV)	Satellite (861.2 ± 0.2 eV)
CoNi-ZIF-67	16.5	13.9	16.8	27.1	17.4	8.3
CoNi-ZIF-67@$Ti_3C_2T_x$	19.5	19.0	12.2	17.9	22.5	8.9

Table S4. Comparisons of OER performance between recent reported CoNi-based electrocatalysts with CoNi-ZIF-67@$Ti_3C_2T_x$.

Catalyst	η_{10} (mV)	Tafel slop (mV dec^{-1})	Electrolyte	Substrate	Mass loading (mg cm^{-2})	Ref.
CoNi-ZIF-67@$Ti_3C_2T_x$	323	65.1	0.1 M KOH	Glassy carbon	0.5	This work
Titanium carbide-CoBDC	410	48.2	0.1 M KOH	Glassy carbon	0.21	1
Co_3O_4/Co_2MnO_4	540	N.A.	0.1 M KOH	Glassy carbon	0.028	2
$Co_{0.13}Ni_{0.87}Se_2$	320	94	1.0 M KOH	Ti plate	1.67	3
$NiCo_2O_4$/CNTs	390	68.1	1.0 M KOH	Glassy carbon	0.2	4
$Ni_xCo_{3-x}O_4$ nanowires	337	75	1.0 M KOH	Glassy carbon	0.7	5
$NiCo_2O_4$ nanoneedles	565	292	1.0 M KOH	FTO glass	0.53	6
NiCo-LDH nanosheets	420	113	0.1 M KOH	Glassy carbon	1.76	7

Table S5. The simulated internal resistance (R1) and charge transfer resistance (R2) from the Nyquist plots in Figure 6b.

	**R1**	**R2**
$Ti_3C_2T_x$	2.69	16.34
CoNi-ZIF-67@$Ti_3C_2T_x$	3.15	11.96
CoNi-ZIF-67	12.08	48.65
IrO_2	6.94	16.21

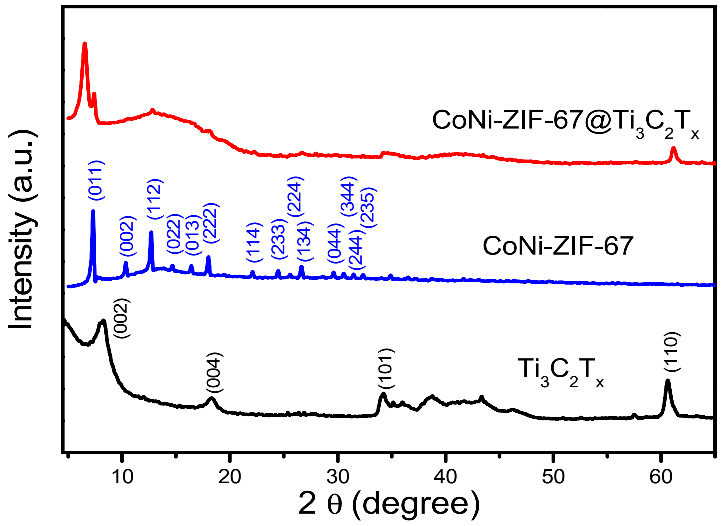

Figure S1. Enlarged image of XRD patterns of catalysts.

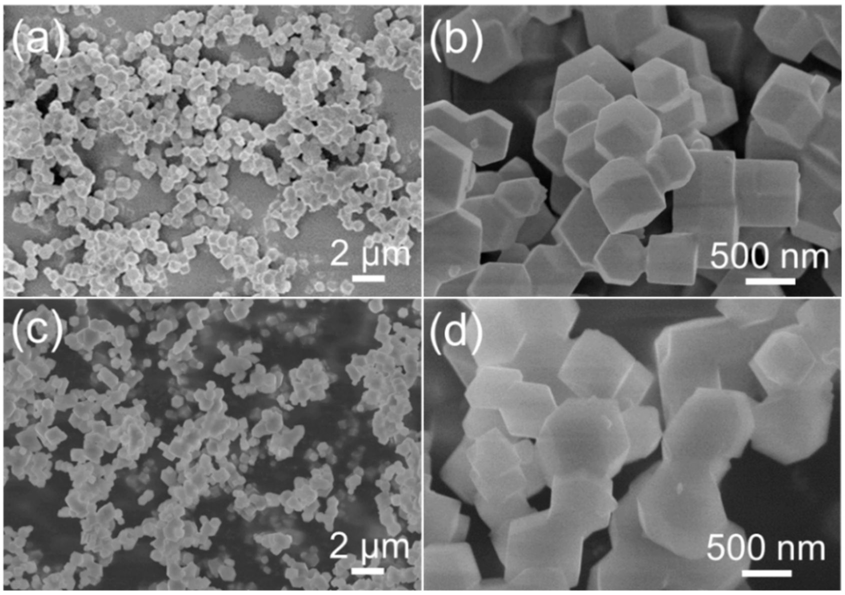

Figure S2. SEM images of pure CoNi-ZIF-67 prepared by the same procedure but with CTAB (**a**,**b**) and without CTAB (**c**,**d**) at different magnifications.

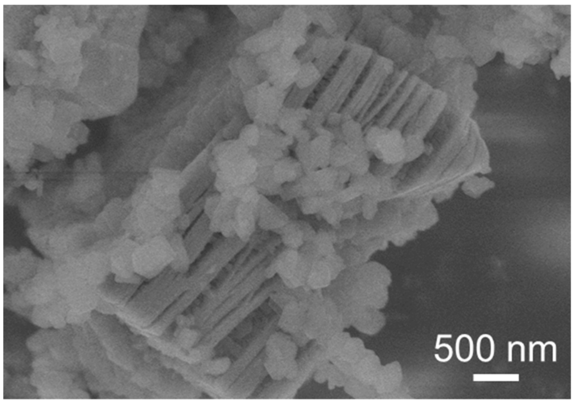

Figure S3. SEM image of CoNi-ZIF-67@$Ti_3C_2T_x$ without using CTAB.

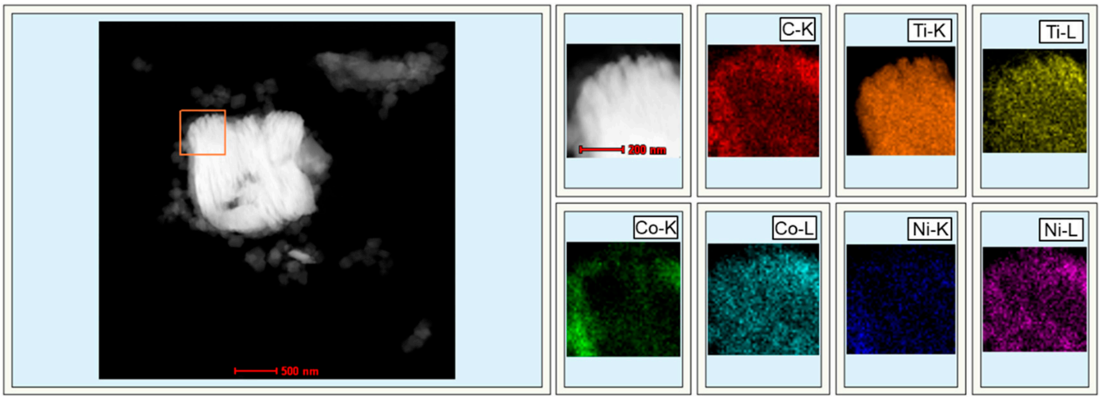

Figure S4. HAADF-STEM images and the corresponding elemental maps of C, Ti, Co and Ni in the CoNi-ZIF-67@$Ti_3C_2T_x$.

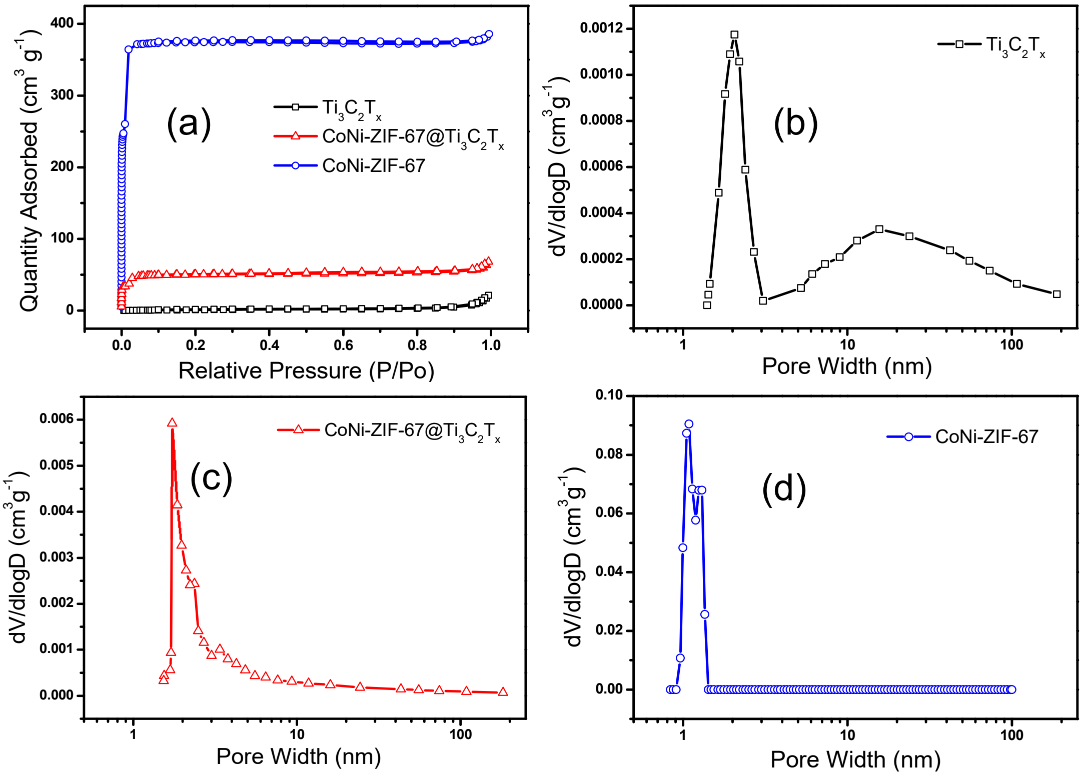

Figure S5. Nitrogen adsorption isotherms and pore size distribution of the pristine $Ti_3C_2T_x$, CoNi-ZIF-67@$Ti_3C_2T_x$ and pure CoNi-ZIF-67.

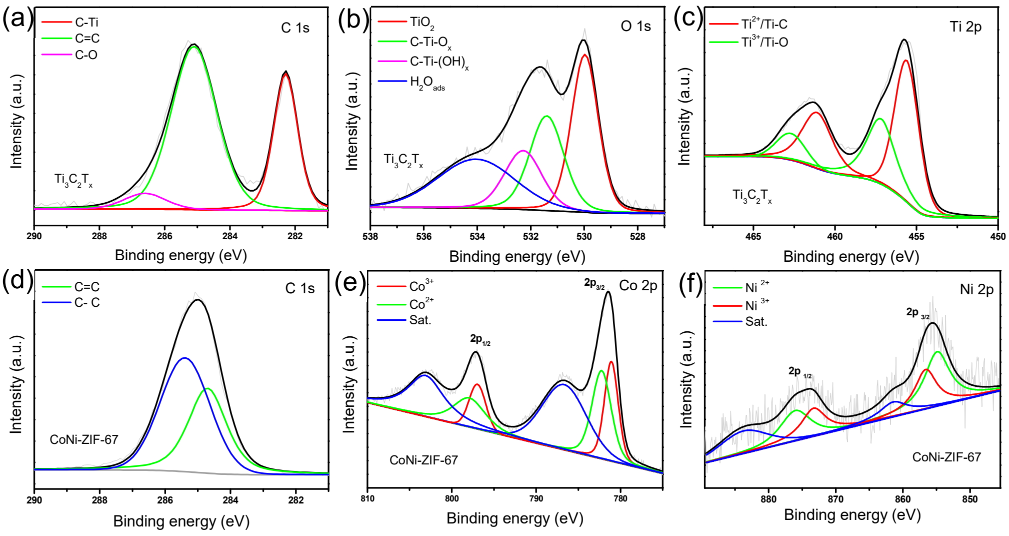

Figure S6. XPS results of $Ti_3C_2T_x$ (a–c) and CoNi-ZIF-67 (d–f).

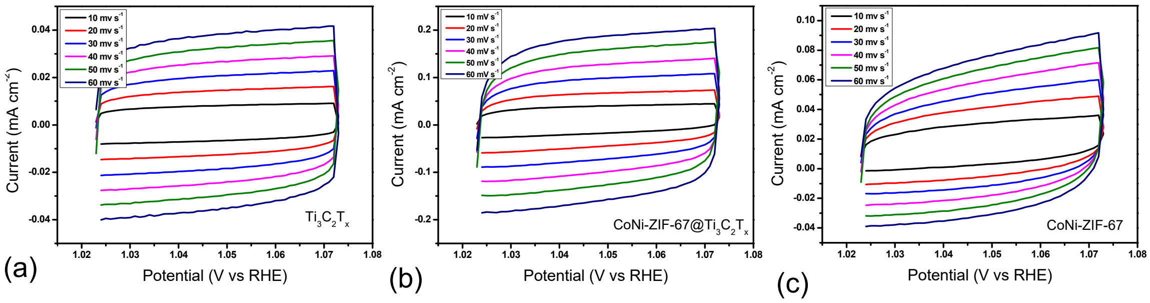

Figure S7. CV curves in a potential range of 1.023-1.073 V vs. RHE of catalysts: $Ti_3C_2T_x$ (**a**), CoNi-ZIF-67@$Ti_3C_2T_x$ (**b**) and pure CoNi-ZIF-67 (**c**).

References

1. Zhao, L.; Dong, B.; Li, S.; Zhou, L.; Lai, L.; Wang, Z.; Zhao, S.; Han, M.; Gao, K.; Lu, M.; Xie, X.; Chen, B.; Liu, Z.; Wang, X.; Zhang, H.; Li, H.; Liu, J.; Zhang, H.; Huang, X.; Huang, W. Interdiffusion reaction-assisted hybridization of two-dimensional metal-organic frameworks and Ti3C2Tx nanosheets for electrocatalytic oxygen evolution. *ACS Nano* **2017**, 11, 5800-5807.
2. Wang, D.; Chen, X.; Evans, D. G.; Yang, W. Well-Dispersed Co_3O_4/Co_2MnO_4 nanocomposites as a synergistic bifunctional catalyst for oxygen reduction and oxygen evolution reactions. *Nanoscale* **2013**, 5, 5312-5315.
3. Liu, T.; Asiri, A. M.; Sun, X. Electrodeposited Co-doped $NiSe_2$ nanoparticles film: a good electrocatalyst for efficient water splitting. *Nanoscale* **2016**, 8, 3911-3915.
4. Cheng, H.; Su, Y.; Kuang, P.; Chen, G.; Liu, Z. Hierarchical $NiCo_2O_4$ nanosheet decorated carbon nanotubes towards highly efficient electrocatalyst for water oxidation. *J. Mater. Chem. A* **2015**, 3, 19314-19321.
5. Yan, X.; Li, K.; Lyu, L.; Song, F.; He, J.; Niu, D.; Liu, L.; Hu, X.; Chen, X. From water oxidation to reduction: transformation from $Ni_xCo_{3-x}O_4$ nanowires to NiCo/$NiCoO_x$ heterostructures. *ACS App. Mater. Inter.* **2016**, 8, 3208-3214.
6. Shi, H.; Zhao, G. Water oxidation on spinel $NiCo_2O_4$ nanoneedles anode: microstructures, specific surface character, and the enhanced electrocatalytic performance. *J. Phys. Chem. C* **2014**, 118 (45), 25939-25946.
7. Jiang, J.; Zhang, A.; Li, L.; Ai, L. nickel-cobalt layered double hydroxide nanosheets as high-performance electrocatalyst for oxygen evolution reaction. J. Power Sources 2015, 278, 445-451.
